# Prognostic modeling in early rheumatoid arthritis: reconsidering the predictive role of disease activity scores

**DOI:** 10.1007/s10067-024-06946-z

**Published:** 2024-03-27

**Authors:** Alix Bird, Lauren Oakden-Rayner, Luke A. Smith, Minyan Zeng, Shonket Ray, Susanna Proudman, Lyle J. Palmer

**Affiliations:** 1 Australian Institute of Machine Learning, University of Adelaide, Corner Frome Road and North Terrace, Adelaide, SA 5000 Australia; 2 School of Public Health, The University of Adelaide, North Terrace, Adelaide, SA 5000 Australia; 3 Artificial Intelligence and Machine Learning, GSK Plc, South San Francisco, CA USA; 4 Department of Rheumatology, Royal Adelaide Hospital, Adelaide, SA 5000 Australia

**Keywords:** Linear regression, Outcome assessment, Patient reported outcome, Precision medicine, Prognosis, Rheumatoid arthritis

## Abstract

**Objective:**

In this prospective cohort study, we provide several prognostic models to predict functional status as measured by the modified Health Assessment Questionnaire (mHAQ). The early adoption of the treat-to-target strategy in this cohort offered a unique opportunity to identify predictive factors using longitudinal data across 20 years.

**Methods:**

A cohort of 397 patients with early RA was used to develop statistical models to predict mHAQ score measured at baseline, 12 months, and 18 months post diagnosis, as well as serially measured mHAQ. Demographic data, clinical measures, autoantibodies, medication use, comorbid conditions, and baseline mHAQ were considered as predictors.

**Results:**

The discriminative performance of models was comparable to previous work, with an area under the receiver operator curve ranging from 0.64 to 0.88. The most consistent predictive variable was baseline mHAQ. Patient-reported outcomes including early morning stiffness, tender joint count (TJC), fatigue, pain, and patient global assessment were positively predictive of a higher mHAQ at baseline and longitudinally, as was the physician global assessment and C-reactive protein. When considering future function, a higher TJC predicted persistent disability while a higher swollen joint count predicted functional improvements with treatment.

**Conclusion:**

In our study of mHAQ prediction in RA patients receiving treat-to-target therapy, patient-reported outcomes were most consistently predictive of function. Patients with high disease activity due predominantly to tenderness scores rather than swelling may benefit from less aggressive treatment escalation and an emphasis on non-pharmacological therapies, allowing for a more personalized approach to treatment.

**Table:** 

**Key Points** *• Long-term use of the treat-to-target strategy in this patient cohort offers a unique opportunity to develop prognostic models for functional outcomes using extensive longitudinal data.* *• Patient reported outcomes were more consistent predictors of function than traditional prognostic markers.* *• Tender joint count and swollen joint count had discordant relationships with future function, adding weight to the possibility that disease activity may better guide treatment when the components are considered separately.*

**Supplementary Information:**

The online version contains supplementary material available at 10.1007/s10067-024-06946-z.

## Introduction

Rheumatoid arthritis (RA) is an inflammatory disease of the synovial joints that often leads to progressive joint damage and disability [1]. The treat-to-target treatment (T2T) approach, wherein disease-modifying anti-rheumatic drug (DMARD) therapy are escalated until a predefined disease activity target has been achieved, has been recommended therapy for more than two decades [2]. Nonetheless, some patients experience ongoing symptoms and loss of function, often with progressive joint damage [3]. Improving the prediction of treatment response and outcomes is vital to guide and optimize treatment decisions [4]. Prior to the T2T era, factors that predicted disability according to a 2003 systematic review included age, female sex, rheumatoid factor positivity, high pain scores, low SES, joint tenderness, and depression [5]. There is currently no consensus regarding prognostic models in patients receiving the T2T approach appropriate for clinical use due to the historically small, demographically uniform cohorts, changing treatment regimens, and lack of external validation [6].

Functional outcomes in RA are frequently measured by the Health Assessment Questionnaire (HAQ), a validated and widely used instrument that was first developed in 1978. It is intended to integrate function as it relates to structural damage, disease activity, pain, and psychosocial factors [7], while the modified HAQ (mHAQ) is an abridged version to improve feasibility of use in practice [8]. We conducted a case series study in a cohort of patients with early RA recruited over the period 1998 to 2021. The clinic was an early adopter of the treat-to-target approach, which poses a unique opportunity to investigate predictors of response to relatively contemporary treatment strategies. Identifying which baseline factors are amenable to treatment lends itself to more personalized treatment approaches. Four multivariable prediction models were developed to investigate the predictive factors of the mHAQ. Each model was internally validated using cross-validation [9].

## Methods

### Study population

The study population comprised patients enrolled in the early RA cohort at the Royal Adelaide Hospital from 1998 to 2021. Consecutive patients diagnosed with RA which met the 1987 [10] or 2010 ACR-EULAR [11] criteria for classification with RA (depending on enrolment year) were eligible. While anti-cyclic citrullinated peptide (ACPA) was only included in the 2010 classification criteria, it has been measured in this cohort in sera stored since its inception.

Patients provided informed consent for the use of their data for research (CALHN HREC approval 120,618), and subsequent approval was obtained for extraction and use of the data in this study (CALHN HREC approval 15,056). Patients were included in this cohort if the onset of symptoms of RA occurred within the preceding 12 months, they were DMARD-naive, and they were over the age of 18 [12]. Patients were managed according to predefined treatment algorithms whereby they commenced triple therapy with methotrexate, sulfasalazine, and hydroxychloroquine. DMARD up-titrations were made according to previously published algorithms [13]. While the treatment protocol changed in 2005 to include the use of biologic DMARDs (bDMARDs) and subsequently targeted synthetic DMARDs (tsDMARDs), the proportion of patients who commenced on DMARDs remained low due to the strict criteria for subsidized therapy in Australia [14]. Data for up to 5 years post-initial diagnosis were included, given the attrition rate in a real-world population. Patients were excluded if they did not have an initial mHAQ score recorded or if data were recorded more than a month after treatment commencement (*n* = 3).

### Outcome

The primary outcome was the mHAQ score, an extensively validated measure of self-reported function [7]. Patients were assessed by a rheumatology nurse trained in metrology, and the treating rheumatologist was blinded to the mHAQ score. The resulting summed raw score ranges from 0 to 24, with a higher score indicating more dysfunction.

### Candidate predictors

We considered several candidate predictors measured at baseline (defined as treatment initiation) based on subject matter knowledge and prior findings in the literature. There was a high degree of collinearity between C-reactive protein (CRP), erythrocyte sedimentation rate (ESR), and the disease activity composite measures such as the simplified disease activity index (SDAI), clinical disease activity index (CDAI), DAS28-CRP, and DAS28-ESR. We elected to include only the components of the DAS28-CRP, which combines the patient-derived or influenced measure of patient global assessment measured on a 100 mm visual analogue scale (VAS) and tender joint count (TJC) and the physician-derived measure of swollen joint count (SJC) and CRP.

DMARD use was included as a potential explanatory variable, categorized as (a) mono- or dual therapy, (b) triple therapy, (c) added leflunomide, or (d) added any other drug (i.e., cyclosporine, gold, b/tsDMARDs). Medications at 1 year were used as this allowed for the greatest separation between patients given all were managed with the same treat-to-target algorithm. As sample sizes were small and the patient cohort was predominantly of European ancestry, ethnicity was coded as a binary variable (“European ancestry” and “non-European ancestry”). Socioeconomic status (SES) was based on postcode data and the Socio-economic Indexes for Areas, which divides postcodes into deciles resulting in a score from 1 to 10 with 1 indicating the most disadvantaged areas and 10 indicating the most advantaged areas [15].

### Statistical analysis

Continuous variables were reported with means and standard deviations (SD) or medians and interquartile ranges (IQR) while categorical variables were reported as percentages and frequencies. Bivariate analyses were based on *t*-tests or Mann–Whitney *U* tests to investigate associations between continuous variables and binary variables. Bivariate associations between continuous variables were investigated using Pearson’s correlation coefficients or Spearman’s ranked correlation coefficient. Associations between two binary variables were investigated using chi-squared tests. Variables that were associated with baseline mHAQ (*p* < 0.10) were chosen to be included in multivariate models to reduce model complexity by limiting the degrees of freedom. The distribution of residuals was analyzed using the Kolmogorov–Smirnov (KS) test to ensure the assumption of normality was not violated for use of a standard GLM.

Four multivariate predictive models were developed that varied in the way the variables and outcomes were used:

1. A generalized linear model (GLM) [16] to predict baseline mHAQ using the variables collected at baseline
2. A GLM (Poisson) predicting mHAQ at 1 year based on variables collected at baseline
3. A GLM (Poisson) predicting mHAQ at 18 months based on variables collected at 6 months post diagnosis
4. A linear mixed effects (LME) [17] longitudinal model to predict serially measured mHAQ based on variables collected contemporaneously

Variables were chosen by backward selection. Model 4 used an *α* of 0.05 given it had a greater a priori power as it took advantage of repeated measures. The Akaike information criteria (AIC) was used for variable selection in the smaller datasets, which corresponded to an *α* of 0.157 [18]. The performance of each model was estimated using tenfold cross-validation, which has been shown to achieve minimal bias [9]. Multiple imputation using chained equations (MICE) was performed on the training segment of the data to impute missing values (50 imputations), and resultant models were pooled using Rubin’s rules [19]. The missing values in the validation data were imputed using the imputation models initially developed on the training data, to avoid train-test contamination. Variables with greater than 10% of missing values were removed to avoid biasing our analysis [20]. The final pooled model was tested on the validation data. The mHAQ was dichotomized at a threshold of 0.25 to construct receiver operator curves (ROC) and investigate discriminative performance. We selected this value as it approximates the minimum clinically important difference identified in previous work [21] and thus could be considered to constitute a threshold for clinically meaningful disability.

The root mean squared error (RMSE) and coefficient of determination (*R*^2^) were calculated for each model. The variables that were significant across the majority of cross-validation folds (greater than 50%) were included in a final model using the entirety of the data, in order to report the variable coefficients and *p* values. Backward selection was conducted again at this point to remove variables with a *p* value above the threshold. Statistical analysis was conducted using Python 3.8.8 [22], statsmodels 0.1 [23], and pyMIDAS [24].

## Results

### Descriptive statistics

**Fig. 1:**
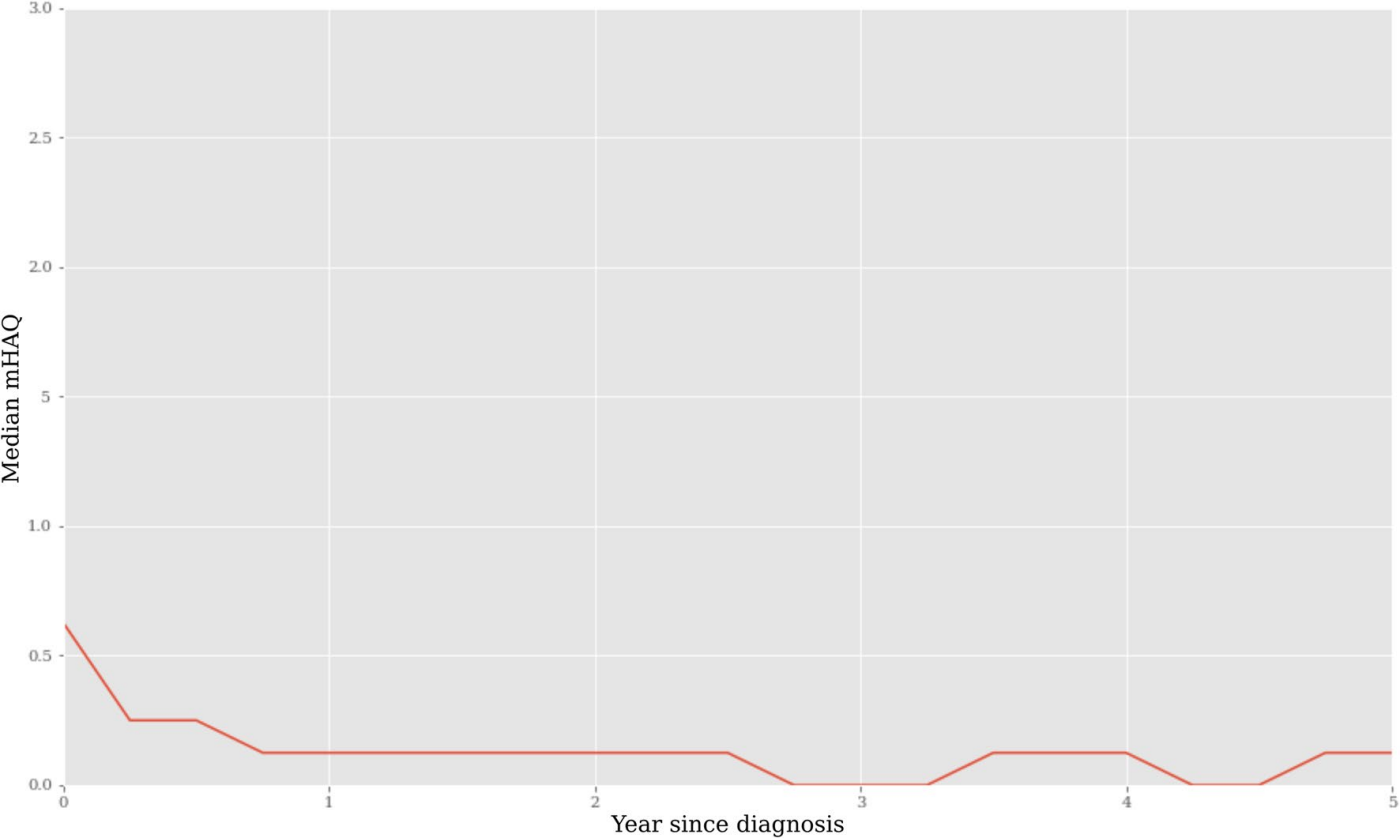
Median mHAQ per year since initial diagnosis

Patients had an initial median mHAQ of 0.6 (IQR 0.3–1.1), which improved to 0.0 (IQR = 0.0–0.375) at 1-year post-diagnosis (Table 1). At baseline, 27.9% of patients had a mHAQ of zero, 95.7% at 1 year and 93.7% at 18 months, indicating significant functional improvement with treatment. The median mHAQ score had a high initial value, with a rapid drop in mHAQ before plateauing for 5 years post-diagnosis (Fig. 1). Additionally, the median reported 5-year post-diagnosis mHAQ per calendar year did not change significantly, despite the introduction of biologics in 2005 (Supplementary Fig. 1). This suggests that treatment efficacy did not change significantly since inception of the cohort (*p* = 0.43).

**Table 1:** Characteristics of study population (*n* = 397)

Variable	Statistic	Missingness	*p* value of relationship with mHAQ at baseline
Sociodemographic factors			
Age at initial appointment	55.2 (15.1)*	0 (0.0%)	0.21
Female sex *n* (%)	273 (68.8%)	0 (0%)	0.85
Socioeconomic status based on postcode	6.0 (3.0–8.0)^#^	20 (5.0%)	0.04
European ancestry	351 (88.4%)	0 (0%)	0.83
Attended university	104 (26.2%)	0 (0%)	0.82
Patient reported outcomes			
HAQ at first appointment	0.6 (0.3–1.1)^#^	0 (0.0%)	N/A
HAQ at 1 year (*n* = 356 patients)	0.1 (0.0–0.4)^#^	0 (0.0%)	0.20
Patient pain (VAS)	60.0 (34.0–74.0)^#^	10 (2.5%)	< 0.001
Early morning stiffness (minutes)	60.0 (20.0–120.0)^#^	10 (2.5%)	< 0.001
Fatigue	53.0 (27.0–70.0)^#^	26 (6.6%)	< 0.001
Patient global assessment (VAS)	50.0 (25.0–69.0)^#^	0 (0%)	< 0.001
TJC	15.0 (8.0–24.0)^#^	4 (1.0%)	< 0.001
Other clinical variables			
Duration of follow-up (weeks)	236 (133.0–252.0)^#^	N/A	< 0.001
Physician global assessment (VAS)	50.0 (30.0–66.0)^#^	19 (4.8%)	< 0.001
SJC	10.0 (5.0–16.0)^#^	4 (1.0%)	< 0.001
CRP	4.9 (4.1–5.6)^#^	9 (2.3%)	< 0.001
BMI^†^	28.5 (6.3)*	22 (10.2%)	0.09
Positive rheumatoid factor	250 (63.0%)	0 (0%)	0.54
Positive anti-CCP *n* (%)	227 (57.2%)	0 (0%)	0.21
Positive shared epitope *n* (%)	249 (62.8%)	0 (0%)	0.68
Fish oil consumption per day	0.0 (0–1.4)^#^	5 (1.3%)	0.20
Medications at 1 year post diagnosis *n* (%)		0 (0%)	
Less than triple therapy	170 (48.3%)		0.33
Triple therapy	150 (42.6%)		0.30
Added leflunomide	28 (8.0%)		0.83
Added another drug	4 (1.1%)		0.84
Comorbidities (self-reported) *n* (%)			
Ever smoked	216 (55.1%)	5 (1.3%)	0.64
Chronic back pain	70 (17.6%)	0 (0%)	0.60
Fibromyalgia	26 (6.6%)	0 (0%)	0.74
Osteoarthritis	178 (44.8%)	0 (0%)	0.27
Depression	91 (22.9%)	0 (0%)	0.34

*Mean (SD)

^#^Median (IQR)

^†^Variable excluded due to missingness > 10%

### Bivariate analysis

**Fig. 2:**
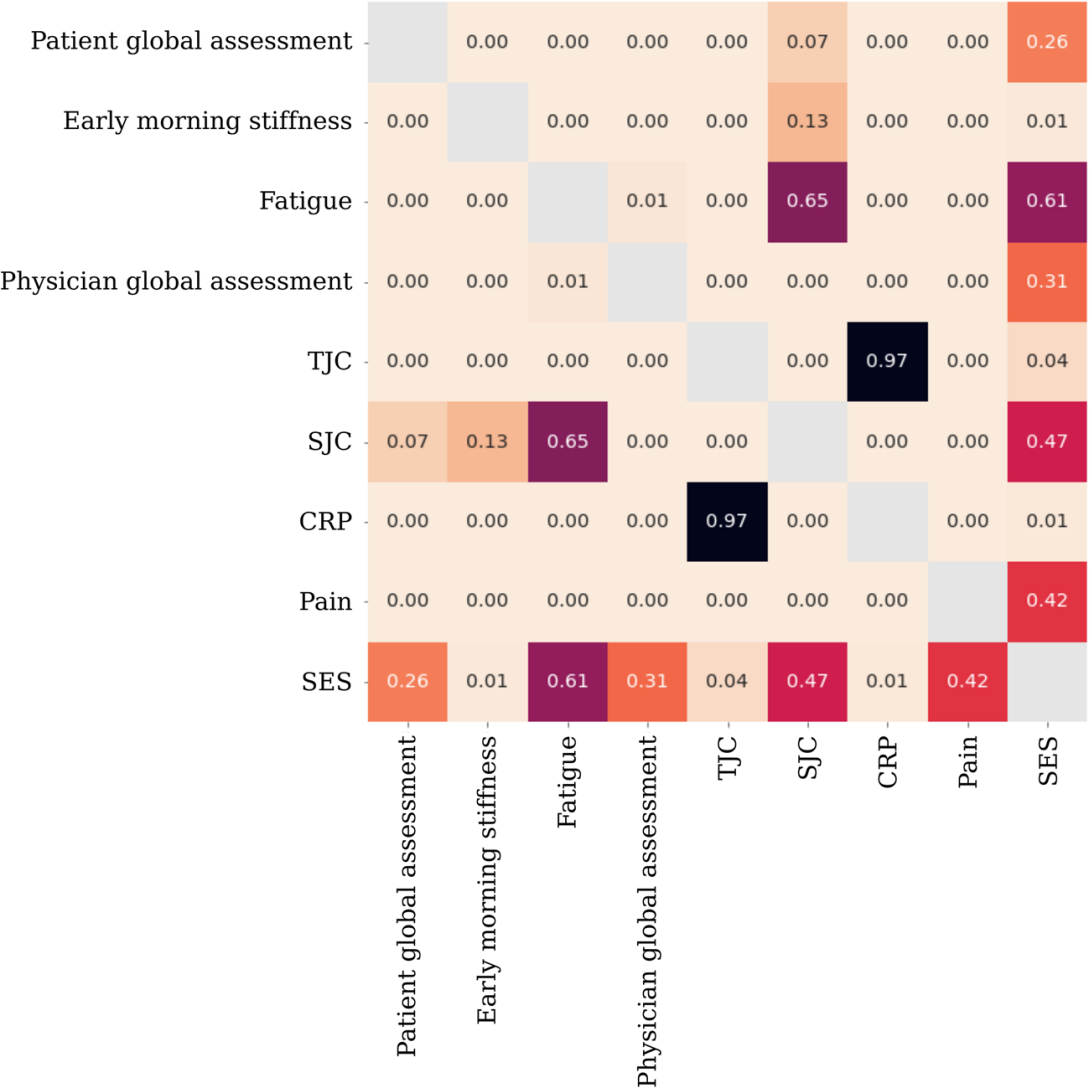
Heatmap demonstrating the *p* value of the correlations between each variable

Bivariate analyses among variables collected at baseline were conducted to identify the variables to include in multivariate models and to investigate for collinearity that was not previously identified (Fig. 2). A total of 25 variables were included as potential predictors in our analyses (Table 1). Variables found to be associated with mHAQ score at baseline were patient global assessment, stiffness, fatigue, physician global assessment, TJC, SJC, BMI, patient pain, CRP, and socioeconomic status (SES) based on postcode.

### GLM predicting baseline HAQ from baseline variables

**Fig. 3:**
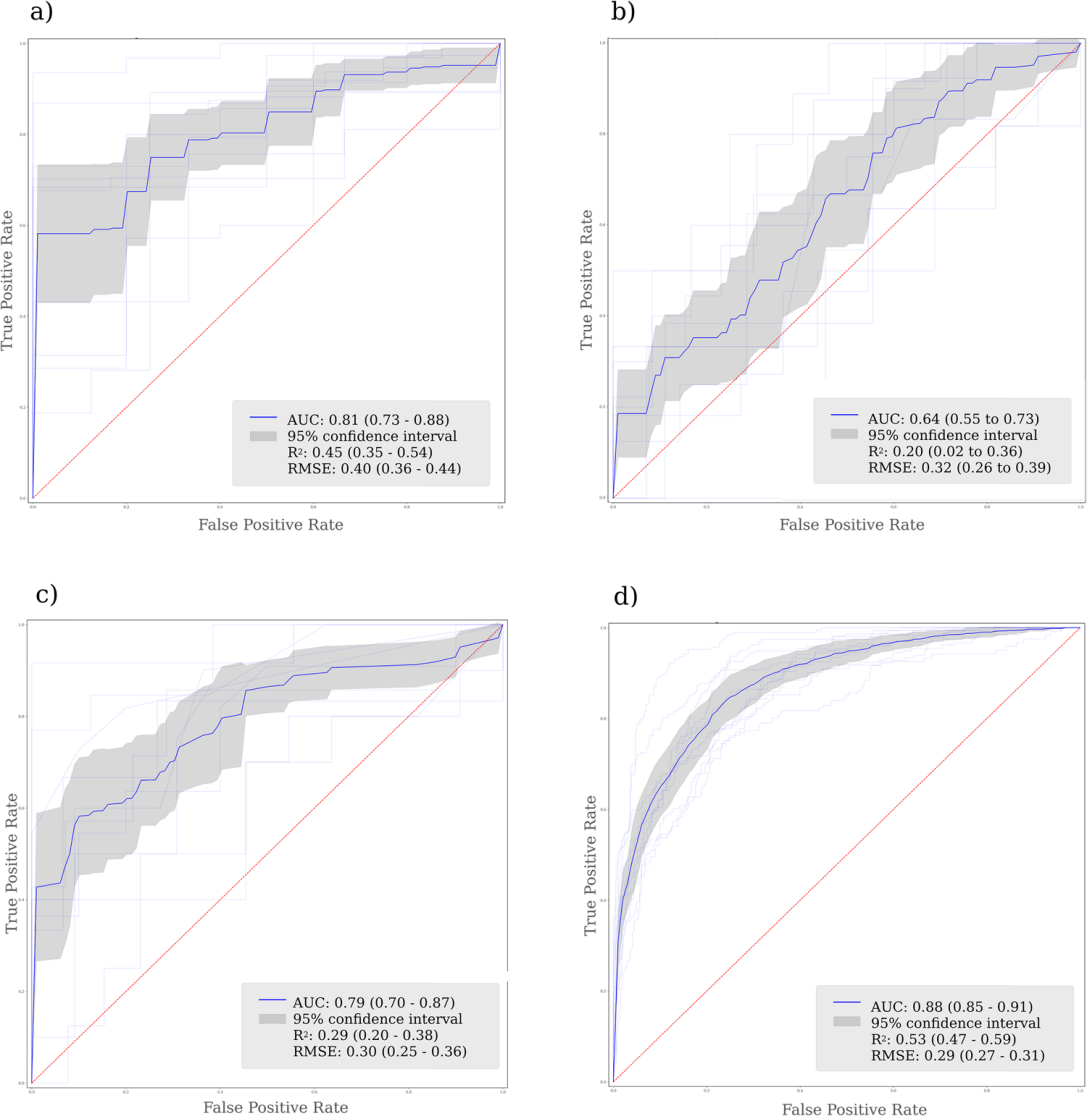
Receiver operator characteristic curve for each model including the AUROC, *R*^2^, and RMSE and their 95% confidence interval. a) GLM to predict baseline mHAQ, b) GLM predicting mHAQ at one year from baseline variables, c) GLM predicting 18-months mHAQ from variables collected at 6-months, d) a LME longitudinal model to predict serially measured mHAQ from variables collected contemporaneously

The KS test was applied to the residuals, with a *p* value of 0.34, suggesting that the sample likely came from a normal distribution and a standard GLM was deemed suitable. A total of 397 patients were included in GLM multivariate modeling. The mean AUC achieved was 0.81 (0.73–0.88), *R*^2^ 0.45 (0.35–0.54), and RMSE 0.40 (0.36–0.44) (Fig. 3). Six variables were found to be associated with increased baseline mHAQ score: increased patient global assessment (*β* = 0.008, *p* < 0.001), duration of morning joint stiffness (*β* = 0.012, *p* = 0.015), physician global assessment (*β* = 0.003, *p* = 0.037), TJC (*β* = 0.008, *p* < 0.001), CRP (*β* = 0.002, *p* = 0.009), and pain (*β* = 0.004, *p* < 0.001) (Table 2).

**Table 2:** Predictive variable for each model, coefficients, and *p* values

Term	Coefficient	*p* value
GLM predicting mHAQ at baseline		
Constant	− 0.168	0.003
Patient global assessment	0.008	< 0.001
TJC	0.008	< 0.001
Pain	0.004	< 0.001
CRP	0.019	0.009
Early morning stiffness	0.012	0.015
Physician global assessment	0.003	0.037
GLM predicting 1-year mHAQ		
Constant	− 1.60	< 0.001
SJC	− 0.409	0.007
TJC	0.449	0.002
Baseline HAQ	0.285	0.044
GLM predicting 18-month mHAQ		
Constant	0.057	0.025
Baseline HAQ	0.617	< 0.001
LMEM predicting serial mHAQ		
Constant	− 0.113	< 0.001
Fatigue	0.001	< 0.001
Physician global assessment	0.001	< 0.001
Patient global assessment	0.003	< 0.001
Duration of morning stiffness	0.007	< 0.001
Pain	0.004	< 0.001
Patient global assessment	0.003	< 0.001
CRP	0.003	< 0.001
TJC	0.004	< 0.001
Baseline HAQ	0.177	< 0.001
Weeks since first visit	− 0.0001	0.001

### GLM predicting 1-year HAQ from baseline variables

A total of 352 patients were included in GLM multivariate modeling. Given a *p* value of < 0.001 when conducting the KS test on the residuals, a Poisson regression was used in this instance. The performance for this model was somewhat lower, with a mean AUC of 0.64 (0.55–0.73), *R*^2^ of 0.20 (0.02–0.36), and RMSE 0.32 (0.26–0.39) (Fig. 3). Baseline mHAQ score (*β* = 0.285, *p* = 0.044), TJC (*β* = 0.449, *p* = 0.002), and SJC (*β* =  − 0.409, *p* = 0.007) were significant predictive variables (Table 2). Baseline mHAQ was a particularly strong predictor, with a one unit increase in baseline mHAQ being associated with a 0.17 unit increase in 1-year mHAQ.

### GLM predicting 18-month HAQ from 6-month variables

A total of 318 patients were included in GLM multivariate modeling. A Poisson regression was again used due to a *p* value of < 0.001 with the KS test. The only variable found to be predictive of 18-month mHAQ was mHAQ measured at 6 months (*β* = 0.617, *p* =  < 0.001) (Table 2). The mean AUC achieved was 0.79 (0.70–0.87), *R*^2^ 0.29 (0.20–0.38), and RMSE 0.30 (0.25–0.36) (Fig. 3).

### Linear mixed effects model

A total of 397 patients with 8264 clinic appointments were used in LME longitudinal analyses. The median length of follow-up was 236 weeks (IQR 133.0–252.0 weeks). The median number of clinical assessments in the cohort was 21 (IQR 14–28). Patients without at least one follow-up appointment were excluded from the analysis. Ten variables were included in the final model (Table 2). Physician global assessment (*β* = 0.001, *p* =  < 0.001), patient global assessment (*β* = 0.003, *p* =  < 0.001), pain (*β* = 0.004, *p* =  < 0.001), CRP (*β* = 0.002, *p* =  < 0.001), TJC (*β* = 0.004, *p* =  < 0.001), baseline mHAQ (*β* = 0.18, *p* =  < 0.001), fatigue (*β* = 0.001, *p* =  < 0.001), and duration of morning stiffness (*β* = 0.007, *p* =  < 0.001) were all positively associated with higher mHAQ score. The number of weeks since the initial visit was initially included as a fixed effect and random slope, as well as an interaction term with the temporal variables. However, it was found that time as a random slope was a redundant parameter, so it was subsequently removed. Weeks as a fixed effect was statistically significant (*β* =  − 0.0001, *p* = 0.001), showing that mHAQ decreased very gradually with time. The performance was highest in this model, with a mean AUC of 0.88 (0.85–0.91), *R*^2^ 0.53 (0.47–0.59), and RMSE 0.29 (0.27–0.31) (Fig. 3).

## Discussion

We conducted an analysis of an early RA cohort receiving DMARDs according to a T2T approach to predict response to treatment in terms of function as measured by the mHAQ score. We developed three GLMs and one LME model to predict current and future mHAQ scores in 397 patients. The performance of the statistical models fitted ranged from an AUC of 0.64 to 0.88, *R*^2^ ranging from 0.2 to 0.53, and RMSE ranging from 0.29 to 0.40 (Fig. 3), with the LME model being the highest performing. These results are comparable to previous work which have reported a range of AUCs from 0.78 to 0.82 with internal validation [6].

The predictive variables identified across all models were also largely consistent with previous work. Initial mHAQ has been the most consistent and strongest predictor across many studies [6], as it was in our analyses. In particular, when predicting function at 18 months, 6-month mHAQ was the only variable selected, demonstrating the value of 6-month mHAQ as a prognostic marker. Early response to DMARDs has frequently been noted as an important prognostic sign [25], which our study confirms. The components of the DAS28-CRP, TJC, patient global assessment, and CRP, were predictive of baseline mHAQ, with higher values predicting greater disability. SJC was not predictive. However, when predicting future mHAQ at 1 year, higher TJC predicted higher future mHAQ, while higher SJC predicted lower future mHAQ. This lends weight to the idea that overt signs of synovitis such as joint swelling could suggest dysfunction that is more readily modifiable with DMARDs, while tender joints alone may indicate symptoms that may not be entirely due to synovitis and hence less responsive to DMARDs. Conversely, CRP and patient global assessment were not associated with future function.

In regard to the longitudinal model, patient-derived factors made up the majority of predictors including pain, early morning stiffness, patient global assessment, TJC, and fatigue, in addition to indicators of inflammation, CRP and SJC, as well as physician global assessment. The duration of disease was also a useful predictor, with longer time since diagnosis suggesting slightly improved function. While previous work identified a “J-shaped” pattern to progression [5], with initial drop followed by slow increase in the HAQ score, at least within a 5-year time frame, this does not appear to be the case in our cohort. The identified variables were almost the same as those found in the model predicting mHAQ at baseline, the only difference being the inclusion of fatigue. Regardless of the duration of illness, the variables that predict mHAQ at the same visit remain consistent.

Female sex and age at baseline have often been identified as significant predictors of the mHAQ score in previous publications, although a recent systematic review found that 5 out of 18 (28%) prior studies did not identify an association between age and HAQ and 6 out of 21 (29%) did not identify an association between female sex and HAQ [26]. Our findings were consistent with prior studies that did not find an association of age and sex with the mHAQ score. Both age and sex were excluded from further multivariate analysis in our study on the basis of initial bivariate analysis. It is also possible that our study lacked statistical power to detect modest associations of age and sex with mHAQ score. Previous studies have identified associations between marginalized ethnic groups and function [27]. European vs non-European ancestry was excluded as a predictive variable from the results of initial bivariate analysis, with no association to the mHAQ identified. Our findings may reflect the small size and heterogeneity of the “non-European ancestry” group in our study cohort.

Common comorbidities such as depression, osteoarthritis, chronic back pain, and fibromyalgia were also not useful predictors, despite prior evidence that comorbidities impact measurement of the mHAQ [28]. However, most studies on comorbidities use a composite measure such as the Charlson Comorbidity Index [29] or the Rheumatic Disease Comorbidity Index [30], which span many more diseases. Additionally, we suspect our cohort suffered from underdiagnosis of comorbidities, particularly for fibromyalgia, in that only 6.6% of patients received a diagnosis, despite the prevalence estimate in RA patients ranging from 18 to 24% [31]. We suspect because we did not have access to a more thorough measurement of comorbidities that the associations were not detected in our baseline bivariate analyses.

RF and ACPA were also not found to be predictors of mHAQ score in the current study, consistent with other studies involving contemporary patient cohorts. A 2018 systematic review found that 8 out of 11 (72.7%) papers did not identify any association between RF and HAQ [26]. The 3 studies that did used data collected between 1979 and 1998 [26]. Similarly, 5 out of 6 (83.3.%) papers found no association between ACPA and HAQ, although the study that found an association used a relatively large dataset (*n* = 1995 patients) with patients recruited from 1990 to 2009 [32]. The changing predictive role of RF and ACPA may be because patients who previously would have progressed quickly now receive prompt diagnosis and treatment and no longer progress to significant dysfunction as was the norm in the past. A recent large observational study of 3251 patients suggested that RF/ACPA positivity might indicate *better* response to therapy [33].

Our work suggests that persistent dysfunction despite contemporary treatment may indicate a need to consider approaches other than continual treatment escalation. Recent work using the same patient cohort as analyzed in the current study has highlighted the role of non-nociceptive pain in disease activity [34]. The authors note that the presence of joint pain in the absence of synovitis (i.e., a high TJC and low SJC) implies other potential mechanisms may explain high disease activity scores such as central sensitization to pain. Identifying patients who are likely to have central sensitization and thus a lesser response to treatment escalation is an important factor in guiding treatment decisions. For example, these patients could be directed down a treatment path that less aggressively escalates therapies and emphasizes alternate approaches such as physical rehabilitation, patient education, and psychosocial interventions [35]. A threshold of disease activity that returns the patient to the conventional treatment path would be important, particularly where there is evidence of synovitis. While there have been discussions about the role of treatment de-escalation in certain patients, this approach would allow that to be done proactively rather than reactively, enabling personalized medicine for RA patients.

Another possibility to further improve the reliability of models to allow for eventual clinical implementation is the use of imaging of disease-related joint damage as a candidate predictor. This has not been possible until recently due to the infeasibility of systematically quantifying structural damage in the clinic. The advent of deep learning has offered methods of automatically identifying patterns in imaging data that may be more predictive than clinical and demographic data. While plain radiographs may not be sufficiently sensitive to subtle changes to improve model performance, MRI and ultrasound are being used with increasing frequency and may offer a way to bolster the ability to identify features of RA. This idea has been studied in osteoarthritis by predicting patient pain from knee X-rays, with promising results [36], but to the best of our knowledge remains unexplored in RA.

Our study has been conducted in line with best practices for prognostic modeling as specified in the TRIPOD guidelines [18]. We handled missing data with multiple imputation, estimated performance with cross-validation, and used backward elimination for feature selection. While we had extensive longitudinal data, the patient group was demographically uniform and of moderate size, limiting the generalizability of our models. Our dataset sizes were sufficient based on the commonly accepted 1 to 10 rule of variables compared to degrees of freedom [37]. Unfortunately, we were only able to conduct internal validation of our models due to the difficulty of accessing replication data. Independent datasets from a diverse range of institutions would allow for more thorough validation of our models. Despite this limitation, our study achieved the goal of identifying relationships between patient factors and function and confirming whether previous findings remained consistent in our cohort.

## Conclusion

Accurate prediction of response to therapy in patients with RA is critical for guiding treatment decisions and offering avenues for precision therapy. Our modeling in a cohort receiving long-term T2T DMARD therapy was mostly consistent with the previously identified variables predictive of function as measured by the mHAQ. However, our study also identified that the components of the DAS28-CRP, TJC and SJC, have conflicting relationships with future function, suggesting that these factors might be better considered separately when guiding treatment decisions.

### Supplementary Information

Below is the link to the electronic supplementary material.

Supplementary file1 (DOCX 38 KB)

## References

[1] Harris ED (1990). Rheumatoid arthritis. Pathophysiology and implications for therapy. N Engl J Med.

[2] Smolen JS, Aletaha D, Bijlsma JWJ (2010). Treating rheumatoid arthritis to target: recommendations of an international task force. Ann Rheum Dis.

[3] Visser H, le Cessie S, Vos K, Breedveld FC, Hazes JMW (2002). How to diagnose rheumatoid arthritis early: a prediction model for persistent (erosive) arthritis. Arthritis Rheum.

[4] Bansback N, Young A, Brennan A, Dixey J (2006). A prognostic model for functional outcome in early rheumatoid arthritis. J Rheumatol.

[5] Scott DL, Smith C, Kingsley G (2003). Joint damage and disability in rheumatoid arthritis: an updated systematic review. Clin Exp Rheumatol.

[6] Archer R, Hock E, Hamilton J, Stevens J, Essat M, Poku E, Clowes M, Pandor A, Stevenson M (2018). Assessing prognosis and prediction of treatment response in early rheumatoid arthritis: systematic reviews. Health Technol Assess.

[7] Fries JF, Spitz P, Kraines RG, Holman HR (1980). Measurement of patient outcome in arthritis. Arthritis Rheum.

[8] Pincus T, Summey JA, Soraci SA, Wallston KA, Hummon NP (1983). Assessment of patient satisfaction in activities of daily living using a modified Stanford Health Assessment Questionnaire. Arthritis Rheum.

[9] Berrar D (2019) Cross-validation. https://www.researchgate.net/profile/Daniel-Berrar/publication/324701535_Cross-Validation/links/5cb4209c92851c8d22ec4349/Cross-Validation.pdf. Accessed 13 Jun 2023

[10] Arnett FC, Edworthy SM, Bloch DA, McShane DJ, Fries JF, Cooper NS, Healey LA, Kaplan SR, Liang MH, Luthra HS (1988). The American Rheumatism Association 1987 revised criteria for the classification of rheumatoid arthritis. Arthritis Rheum.

[11] Aletaha D, Neogi T, Silman AJ (2010). 2010 Rheumatoid arthritis classification criteria: an American College of Rheumatology/European League Against Rheumatism collaborative initiative. Arthritis Rheum.

[12] Benjamin O, Goyal A, Lappin SL (2022). Disease Modifying anti-rheumatic drugs (DMARD).

[13] Proudman SM, Keen HI, Stamp LK (2007). Response-driven combination therapy with conventional disease-modifying antirheumatic drugs can achieve high response rates in early rheumatoid arthritis with minimal glucocorticoid and nonsteroidal anti-inflammatory drug use. Semin Arthritis Rheum.

[14] Pharmaceutical Benefits Scheme (PBS). In: Australian Government Department of Health and Aged Care. https://www.pbs.gov.au/pbs/industry/listing/elements/pbac-meetings/psd/2009-12/pbac-psd-bdmards-dec09. Accessed 24 Oct 2023

[15] of Statistics AB (2018) Socio-economic indexes for areas. SEIFA provides measures of socio-economic conditions by geographic area

[16] Nelder JA, Wedderburn RWM (1972). Generalized linear models. J R Stat Soc Ser A.

[17] Pinheiro JC, Bates DM, Pinheiro JC, Bates DM (2000). Linear mixed-effects models: basic concepts and examples. Mixed-effects models in S and S-PLUS.

[18] Collins GS, Reitsma JB, Altman DG, Moons KGM (2015). Transparent reporting of a multivariable prediction model for individual prognosis or diagnosis (TRIPOD): the TRIPOD statement. BMJ.

[19] Rubin DB (2004). Multiple imputation for nonresponse in surveys.

[20] Bennett DA (2001). How can I deal with missing data in my study?. Aust N Z J Public Health.

[21] Wells GA, Tugwell P, Kraag GR, Baker PR, Groh J, Redelmeier DA (1993). Minimum important difference between patients with rheumatoid arthritis: the patient’s perspective. J Rheumatol.

[22] Van Rossum G, Drake FL (1995) Python reference manual. http://www.cs.cmu.edu/afs/cs.cmu.edu/project/gwydion-1/OldFiles/OldFiles/python/Doc/ref.ps. Accessed 21 Jul 2023

[23] Seabold S, Perktold J (2010) Statsmodels: econometric and statistical modeling with python. Proceedings of the 9th Python in Science Conference. 10.25080/majora-92bf1922-011

[24] Bhatia S, Hooi B, Yoon M, Shin K, Faloutsos C (2020). Midas: microcluster-based detector of anomalies in edge streams. AAAI.

[25] Verstappen SMM, van Albada-Kuipers GA, Bijlsma JWJ, Blaauw AAM, Schenk Y, Haanen HCM, Jacobs JWG, Utrecht Rheumatoid Arthritis Cohort Study Group (SRU) (2005). A good response to early DMARD treatment of patients with rheumatoid arthritis in the first year predicts remission during follow up. Ann Rheum Dis.

[26] Gwinnutt JM, Sharp CA, Symmons DPM, Lunt M, Verstappen SMM (2018). Baseline patient reported outcomes are more consistent predictors of long-term functional disability than laboratory, imaging or joint count data in patients with early inflammatory arthritis: a systematic review. Semin Arthritis Rheum.

[27] O’Brien J, Park SH, Blachley T, Marchese M, Middaugh N, Wittstock K, Harrold LR (2024). Disparities in burden of disease in patients with rheumatoid arthritis across racial and ethnic groups. Clin Rheumatol.

[28] Radner H, Smolen JS, Aletaha D (2010). Impact of comorbidity on physical function in patients with rheumatoid arthritis. Ann Rheum Dis.

[29] Charlson ME, Pompei P, Ales KL, MacKenzie CR (1987). A new method of classifying prognostic comorbidity in longitudinal studies: development and validation. J Chronic Dis.

[30] England BR, Sayles H, Mikuls TR, Johnson DS, Michaud K (2015). Validation of the rheumatic disease comorbidity index. Arthritis Care Res.

[31] Zhao SS, Duffield SJ, Goodson NJ (2019). The prevalence and impact of comorbid fibromyalgia in inflammatory arthritis. Best Pract Res Clin Rheumatol.

[32] Humphreys JH, Verheul MK, Barton A, MacGregor AJ, Lunt M, Toes RE, Symmons DP, Trouw LA, Verstappen SM (2016). Anticarbamylated protein antibodies are associated with long-term disability and increased disease activity in patients with early inflammatory arthritis: results from the Norfolk Arthritis Register. Ann Rheum Dis.

[33] Pope JE, Movahedi M, Rampakakis E, Cesta A, Sampalis JS, Keystone E, Thorne C, Bombardier C (2018). ACPA and RF as predictors of sustained clinical remission in patients with rheumatoid arthritis: data from the Ontario Best practices Research Initiative (OBRI). RMD Open.

[34] Pisaniello HL, Whittle SL, Lester S, Menz F, Metcalf R, McWilliams L, Hill CL, Proudman S (2022). Using the derived 28-joint disease activity score patient-reported components (DAS28-P) index as a discriminatory measure of response to disease-modifying anti-rheumatic drug therapy in early rheumatoid arthritis. BMC Rheumatol.

[35] Christie A, Jamtvedt G, Dahm KT, Moe RH, Haavardsholm EA, Hagen KB (2007). Effectiveness of nonpharmacological and nonsurgical interventions for patients with rheumatoid arthritis: an overview of systematic reviews. Phys Ther.

[36] Pierson E, Cutler DM, Leskovec J, Mullainathan S, Obermeyer Z (2021). An algorithmic approach to reducing unexplained pain disparities in underserved populations. Nat Med.

[37] Harrell FE (2001) Regression modeling strategies. Springer Series in Statistics

