# Supplementary material for: Prognostic modeling in early rheumatoid arthritis: reconsidering the predictive role of disease activity scores
Source: Clin Rheumatol. 2024 Mar 27;43(5):1503–12. doi: 10.1007/s10067-024-06946-z (PMC11018671; doi:10.1007/s10067-024-06946-z)

**Figure 1** | Median mHAQ 5 years post treatment initiation across patients per quarter, demonstrating that median outcome has not significantly changed over time (p = 0.43).


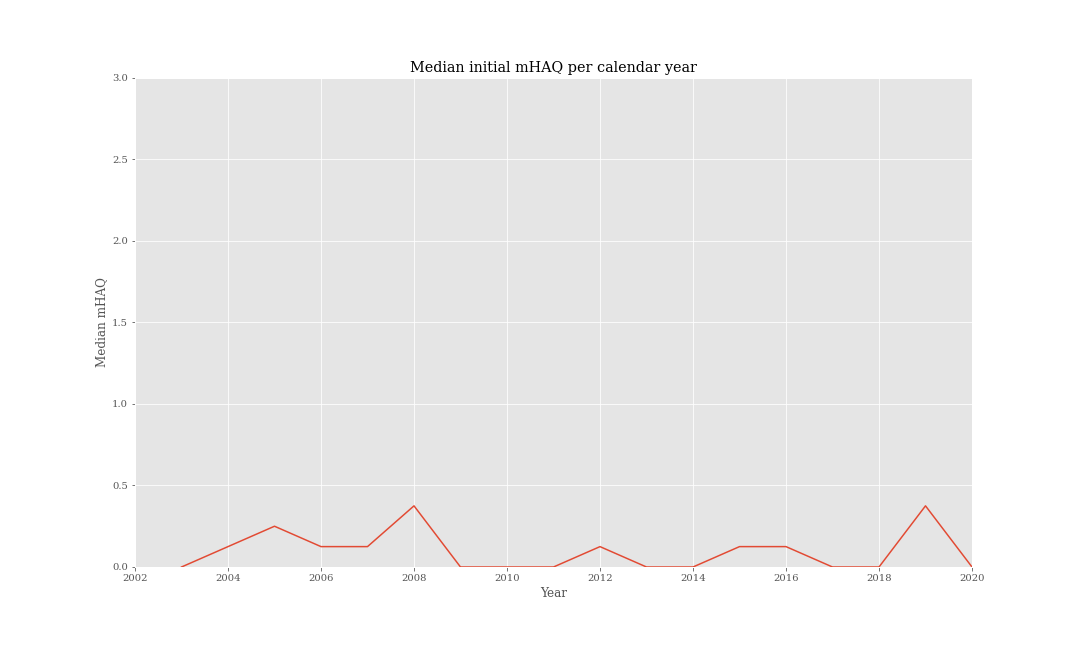

Supplement: Supplementary file 1 — Supplementary file1 (DOCX 38 KB) [file 10067_2024_6946_MOESM1_ESM.docx]
